# Preoperative adjuvant transarterial chemoembolization cannot improve the long term outcome of radical therapies for hepatocellular carcinoma

**DOI:** 10.1038/srep41624

**Published:** 2017-02-03

**Authors:** Lei Jianyong, Zhong Jinjing, Yan Lunan, Zhu Jingqiang, Wang Wentao, Zeng Yong, Li Bo, Wen Tianfu, Yang Jiaying

**Affiliations:** 1 Department of Liver Surgery, West China Hospital of Sichuan University, Chengdu 610041, China; 2 Thyroid and Parathyroid Surgery Center, West China Hospital of Sichuan University, Chengdu 610041, China; 3 Department of Pathology, West China Hospital of Sichuan University, Chengdu 610041, China; 4 Transplantation Center, West China Hospital of Sichuan University, Chengdu 610041, China

## Abstract

Combinations of transarterial chemoembolization (TACE) and radical therapies (pretransplantation, resection and radiofrequency ablation) for hepatocellular carcinoma (HCC) have been reported as controversial issues in recent years. A consecutive sample of 1560 patients with Barcelona Clinic Liver Cancer (BCLC) stage A/B HCC who underwent solitary Radiofrequency ablation (RFA), resection or liver transplantation (LT) or adjuvant pre-operative TACE were included. The 1-, 3- and 5-year overall survival rates and tumor-free survival rates were comparable between the solitary radical therapy group and TACE combined group in the whole group and in each of the subgroups (RFA, resection and LT) (P > 0.05). In the subgroup analysis, according to BCLC stage A or B, the advantages of adjuvant TACE were also not observed (P > 0.05). A Neutrophil-lymphocyte ratio (NLR) more than 4, multiple tumor targets, BCLC stage B, and poor histological grade were significant contributors to the overall and tumor-free survival rates. In conclusions, our results indicated that preoperative adjuvant TACE did not prolong long-term overall or tumor-free survival, but LT should nevertheless be considered the first choice for BCLC stage A or B HCC patients. Radical therapies should be performed very carefully in BCLC stage B HCC patients.

Hepatocellular carcinoma (HCC), the fifth most common malignant tumor worldwide, is the third most common tumor resulting in death^1^. International consensus regarding a common treatment strategy for patients with HCC has not been attained because radical therapies, including resection, liver transplantation and radiofrequency ablation (RFA), are applicable in only 30–40% of patients with HCC, according to the commonly used algorithms, with the majority of patients requiring different approaches^2^. Liver transplantation (LT) should be considered the first choice for these early-stage liver cancer cases in the absence of an extrahepatic target; however, the shortage of liver grafts from deceased donors, as a result of recently decreasing organ donorship and the high risks, including the donor’s death, has limited the development of liver transplantation methodologies^3^. Fortunately, hepatic resection and local ablation therapies have also served as curative therapies for early-stage patients^4^. Treatment outcomes for HCC patients are affected by multiple variables, including tumor burden, the Child-Pugh score of liver function reserve, the performance status of the patient, and preoperative adjuvant therapies^5^.

Transarterial chemoembolization (TACE) is an effective regional therapy that has widely been used since the 1980s for unresectable HCC. Complete necrosis was previously observed in only 30% to 64% of patients with HCC who received TACE before resection^6^. At the same time, even with resectable HCCs, some researchers^7^,^8^,^9^,^10^ reported that TACE might reduce the viability of HCC cells before radical surgery and thus reduce postoperative tumor recurrence. However, others^11^,^12^,^13^,^14^ failed to show any significant survival benefits. Therefore, the role of preoperative TACE for HCC has remained a controversial issue, particularly for early- or intermediate-stage HCC.

In the present study, we attempted to evaluate the effectiveness of preoperative TACE for BCLC stage 0-A or stage B HCCs, and we compared its effectiveness in combination with three radical therapies (RFA, resection, LT) for Barcelona Clinic Liver Cancer (BCLC) stage A or B HCCs.

## Materials and Methods

### Patients and study design

Between January 2002 and May 2008, 1560 consecutive patients who were diagnosed with HCCs at West China Hospital were included in our study. The ethical conduct of this study was approved by our departmental review board (West China Hospital of Sichuan University) in agreement with the 1990 Declaration of Helsinki and subsequent amendments, meanwhile, all patients have signed informed consent. The main inclusion/exclusion criteria are shown in Table 1. All of these patients were divided into a combined TACE and radical therapy group or a simple radical therapy group. The combined TACE and radical therapy group included the TACE plus RFA group (81 cases), TACE plus resection group (268 case), and TACE plus LT group (78 cases), and the solitary radical therapy included the RFA group (163 cases), resection group (633 cases), and LT group (337 cases). All patients in the TACE group received one session of TACE, and radical therapies followed in at least two weeks with liver function recovery; the decision to perform TACE prior to radical therapies was made mainly by the attending physician: destoryed liver function, waiting for the liver graft, hesitation of choice. Liver transplantation was considered the primary treatment for all cases meeting the Milan criteria^15^ or UCSF criteria^16^. The diagnosis of HCC was made based on a positive serum fetoprotein level (>400 ng/ml) with positive imaging findings or at least two enhanced imaging techniques (ultrasound, CT or MRI) showing characteristic findings of arterial hypervascularization in all or some part of the tumor and washout in the portal-venous phase in high-risk patients^17^,^18^, meanwhile. The CT or MRI diagnosis of HCC was based on the presence of lesions with different echogenicity, i.e., hypoechoic, hyperechoic, isoechoic, or a mixed pattern, compared with that of the surrounding liver parenchyma, all of the diagnosis of the HCC were confirmed in preoperative tissue sampling and postoperative histological confirmation. The lesions were examined for tumor size and number, histologic differentiation, and the presence of microvascular and perineural invasion by histological examination.

### Transarterial chemoembolization

All of the TACE procedures in our center were performed by one of three interventional radiologists who had at least 10 years of experience in interventional radiology (LWS, LX or NZY). Depending on the tumor size, location and arterial supply of the tumor, a 3 Fr microcatheter (Microferret; Cool, Bloomington, IN, USA) was advanced toward the tumor-feeding arteries for selective embolization, and TACE of the feeding arteries was performed through further super-selective catheterization as close to the tumor as possible. A mixture of doxorubicin hydrochloride (Adriamycin; Ildong Co., Ltd., Seoul, Korea) and an emulsion of iodized oil (Lipiodol; Laboratorie Guerbet, Aulnay Sous Bois, France) was used for chemoembolization. The dose of the embolization agent was determined according to the tumor size, tumor number, feeding vessels and liver function status. After embolization, angiography was performed to determine the extent of vascular occlusion and to assess the blood flow in other arterial vessels. In our study, the TACE combined group was defined as TACE scheduled for HCC patients on Tuesday, Thursday and Saturday, followed by radical therapy at least 2 weeks later.

### Liver transplantation

Living donor liver transplantation (LDLT) or deceased donor liver transplantation (DDLT) was performed for the patients. All of the LT procedures were performed for the HCC patients in our study using the classic orthotopic method, and the surgical details of the donors’ and recipients’ LDLT or DDLT were discussed in our previous studies^3^,^19^. Each organ donation or transplantation in our center was performed strictly under the guidelines of the Ethical Committee of our hospital, the regulations of the Organ Transplant Committee of Sichuan Province and the Declaration of Helsinki. No prisoners served as donors in our center. For LDLT, the donation was voluntary and altruistic, and we informed the donors and their families of the possible risks of donor hepatectomy. Written consent was provided by the donors for their information to be stored in the hospital database and used for research. The Pre- and Post-operative medication therapy of the patients has been introduced in a prior publication^20^.

### Resection

All of the surgical procedures were performed under general anesthesia and ultrasound guidance. Partial hepatectomy was performed as anatomical resection according to Couinaud, with non-anatomical or wedge resection and a combination of anatomical and non-anatomical resections with or without the “Pringle” maneuver, selective vascular clamping, or selective vascular occlusion. During surgery, parenchymal dissection was performed using an ultrasonic surgical aspirator. Connected tissues, such as neural fibers, adhering around the vessels were grasped instead of directly pinching the vessels. When necessary, the liver pedicle was intermittently clamped in cycles of 10 min of clamping and 5 min of reperfusion. In cases of bleeding, the surgeon gently pressed the bleeding point with the fingers and then dissected around the vessel to obtain a wide operative field. All of the cases were first encouraged to accept liver resection if possible and then RFA or LT and so on. Disease was judged to be unresectable, based on bilobar distribution of lesions, involvement of major vascular structures precluding curative resection, or inadequate hepatic reserve to undergo resection.

### Radiofrequency ablation technique

RFA was performed under ultrasonographic guidance, with the patient under general anesthesia. RFA was performed percutaneously for patients with small or medium tumors in the liver parenchyma, by a laparoscopic approach for patients with small or medium tumors on the liver surface, and through a laparotomy for other circumstances, including patients with tumors proximal to major vascular structures and with large tumors. Tumor ablation was performed by multiple overlapping insertions of a single electrode or three electrode clusters with a 3 cm exposed tip (ValleLab, Burlington, MA, USA). Radiofrequency current was emitted for 12 or 15 min by a 200 W generator set to deliver maximum power with the automatic impedance control method. To maintain the temperature of the electrode tip at less than 20 °C, ice-cold physiological saline was continuously circulated through a cooling catheter connected to the electrode by a peristaltic pump (Watson Marlow; Wilmington, MA, USA). For tumors no larger than 3 cm in diameter, a single electrode was deployed into the center of the tumor. Each application of RFA energy lasted for 10–20 minutes to gain a 5 cm ablation zone. For medium tumors (3.1–5 cm), multiple overlapping zones of ablation were needed for the destruction of the tumor and of a surrounding rim of nontumorous liver. For tumors larger than 5 cm, more multiple overlapping zones of ablation were needed. For patients with more than one lesion, the tumors were ablated separately. To prevent bleeding and tumor seeding, track ablation was performed when withdrawing the RFA electrode in all of the patients. The end point was complete ablation of the visible tumor and at least a 1.0 cm margin of normal liver parenchyma surrounding the tumor.

### Definitions of BCLC stage A and HCCs

BCLC stage A: one to 3 nodules, with none larger than 3 cm in diameter, Child-Pugh class A-B and PS 0.BCLC stage A also included BCLC stage 0 in our study, with solitary targets and no diameter larger than 2 cm (Child A, PST 0); BCLC stage B: 2 to 3 lesions, of which at least 1 was more than 3 cm in diameter, or more than 3 lesions of any diameter, with no extra-hepatic metastasis or macrovascular invasion (segmental branches, right/left and main portal vein, hepatic vein, superior mesenteric vein, inferior vena cava).

### Follow-up and assessment

The overall survival and tumor-free survival rates were major end points, with comparisons between the combined treatment group and the solitary radical therapy group, and the secondary endpoints were procedure-related complications. The efficacy of the radical therapies was evaluated 1 month later by contrast-enhanced computed tomography (CT) or magnetic resonance imaging (MRI) and tumor markers (AFP) and every 2 to 3 months thereafter by experienced liver surgeons and radiologist; to assess the treatment outcome, chest radiography and bone scintigraphy were performed when extrahepatic HCC recurrences were suspected. Time to recurrence was defined as the interval between surgery and the first confirmed recurrence. Postoperative complications were classified using the Clavien system. The overall follow-up time was defined as the interval between the first radical therapy and either local tumor progression or the last follow-up. Patients were followed until death, surgical resection or liver transplantation, or the end date of this study.

### Statistical analysis

The baseline characteristics of the patients are expressed as the means ± standard deviations of the values. For univariate analysis, we used Student’s test for continuous variables, while the Chi-square test or Fisher’s exact test was used to compare categorical variables. Overall survival and tumor-free survival rate were calculated using the Kaplan-Meier method and were compared using the log-rank test. The data were analyzed using univariate and multivariate analyses. Cox proportional hazard models were used for multivariate analysis of factors that were considered significant on univariate analysis. The inclusion of variables into the final models was based on both biological and statistical considerations. The statistical analysis was performed using the Statistical Package for the Social Sciences (SPSS, Inc., Chicago, IL, USA) software (version 17.0). Two-sided P values were computed, and a difference of P < 0.05 was adopted as the threshold for statistical significance.

### Comprehensive Literature review

We comprehensively searched the MEDLINE database using the following medical subject heading (MeSH) terms: hepatocellular carcinoma and liver resection or hepatic resection or transplantation or ablation radiofrequency. Manual searching of relevant references and review articles was also performed. The searched studies were included in our review if they were published in English, compared the efficacy of combined TACE and RFA, LT or resection with a single radical therapy and were published in recent years, to ensure comparability with our retrospective clinical study. Studies involving fewer than 20 patients or recurrence of HCC or those that were not consistent with our inclusion criteria were excluded from the review analysis.

## Results

### Baseline and tumor characteristics

From January 2002 to March 2008, 6788 patients from West China with hepatic malignancies were enrolled in the analysis. Based on the inclusion and exclusion criteria, 1560 cases (23%) were enrolled in the retrospective study. The baseline characteristics of the solitary radical therapy group and TACE combined group are shown in Table 2. The patients’ ages, sexes, races, BMIs, underlying liver diseases, pre-operative anti-viral therapies, and hemoglobin and platelet levels did not show any differences between the solitary radical groups and the combined TACE group. The TACE combined with LT group in our study showed much worse liver function (more Child class B or C patients) than the solitary LT group (P = 0.002), but there were no differences between the RFA and TACE+ RFA groups or between the resection and TACE+ resection group. Neutrophil-lymphocyte ratio (NLR), tumor size and number, alpha fetoprotein (AFP) level, and BCLC stage were the five indices that were used to compare the tumor characteristics, and no significant differences were found between the solitary radical groups and the combined TACE group or among the three subgroups.

### TACE toxicity

Toxicity data for TACE were graded according to the World Health Organization criteria; most of the TACE treatments were well tolerated. The most significant toxicities associated with TACE were transient hepatic toxicity/hepatic function destruction in 366 cases (85.7%), and most of these cases (337, 92.1%) were minor (grade 1); nausea/emesis (232, 54.3%), pain in the upper quadrant (225, 52.7%) and fever (203, 47.5%) followed. A grade 3 adverse reaction developed in 21 of 427 patients (4.9%), and grade 4 adverse reactions occurred in 3 patients (0.7%), as shown in Table 3.

### Operative variables and perioperative outcomes

As shown in Table 4, in the TACE combined with resection group, the intraoperative blood loss was 357.8 ml, which was much lower than that observed in the solitary resection group, with an average of 384.0 ml of blood loss; however, this difference did not reach a statistically significant difference (P = 0.084). Further, a difference between the solitary radical therapy group and the combined TACE group was not observed in the RFA or LT group. At the same time, the mean operative time in the TACE combined with resection group was much shorter than in the solitary resection group (3.9 vs. 4.3, P < 0.001), but no difference was observed in the RFA and LT subgroups. Due to pre-operative TACE, significantly more patients had perihepatic adhesions in the TACE combined group than in the solitary radical therapy group in all three subgroups (P < 0.001). The post-radical therapy complications were graded using the Clavien system, and the overall complications in the RFA and LT subgroups did not show statistically significant differences between the solitary radical groups and the combined TACE group. However, in the resection subgroup, the TACE combined with resection group showed a much higher complication rate than that in the solitary resection group (36.9% vs. 29.4%, P = 0.014), and the combined resection group showed many more complications, primarily biliary leakage(15.3% vs. 8.8%, P = 0.004), although most were classified as grade I or II. The adverse complications (III or IV) were also comparable in the RFA subgroup (P = 0.466), resection subgroup (P = 0.088) and LT subgroup (P = 0.393). When we considered the histological grading from the specimens, no differences between the solitary radical groups and the combined TACE group or any subgroups were found.

### Overall and disease-free survival rates

**Figure 1:**
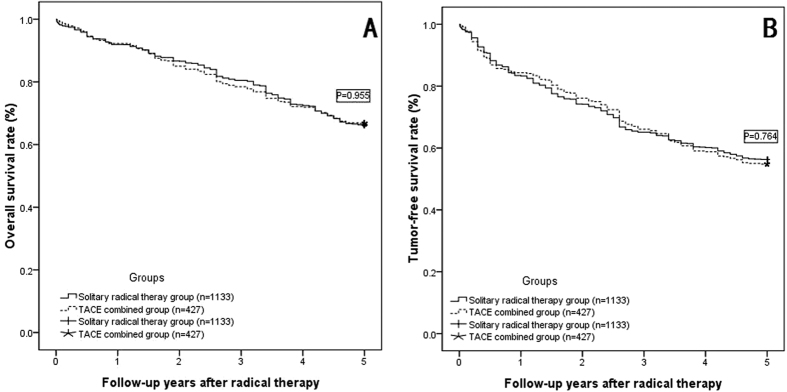
The overall survival rate (OSR) and tumor-free survival rate (TFSR) comparison. (**A**) OSR comparison between solitary radical therapy group and TACE combined group: The overall 1-, 3-, and 5-year survival rates were 92.0%, 80.5%, and 66.0%, respectively, in the solitary radical therapy group (RFA, resection and LT, 1133 cases) and 92.3%, 78.5%, and 66.7% in the TACE combined group (TACE combined with RFA, TACE combined with resection and TACE combined with LT, 427 cases) (P = 0.955); (**B**) TFSR comparison between two groups: The tumor-free survival rates were 83.4%, 65.1%, and 56.3%, respectively, in the solitary radical therapy group and 84.3%, 66.3%, and 54.8% in the TACE combined group (P = 0.746).

For the total of 1560 patients, we divided these patients into two groups: the solitary radical therapy group (1133cases) and the TACE combined group (427 cases); no significant differences were observed in either overall survival rate (OSR) or tumor-free survival rate (TFSR) between the groups (as shown in Table 5). The overall 1-, 3-, and 5-year survival rates were comparable between the solitary radical therapy group and the TACE combined group (shown in Fig. 1A, P = 0.955), the tumor-free survival rates were also comparable between two groups (shown in Fig. 1B, P = 0.746).

**Figure 2:**
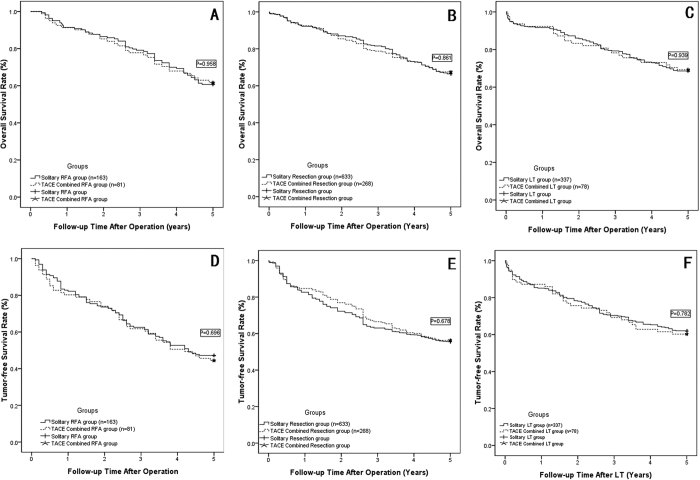
The OSR and TFSR comparison beween the solitary radical therapy group and TACE combined radical therapy group. (**A**) OSR comparison between the solitary RFA group and TACE combined with RFA group: two groups showed comparable OSR (P = 0.958); (**B**) OSR comparison between the solitary Resection group and TACE combined with Resection group: two groups showed comparable OSR (P = 0.861); (**C**) OSR comparison between the solitary LT group and TACE combined with LT group: two groups showed comparable OSR (P = 0.939); (**D**) TFSR comparison between the solitary RFA group and TACE combined with RFA group: two groups showed comparable OSR (P = 0.696); (**E**) TFSR comparison between the solitary Resection group and TACE combined with Resection group: two groups showed comparable OSR (P = 0.678); (**F)** TFSR comparison between the solitary LT group and TACE combined with LT group: two groups showed comparable OSR (P = 0.782).

The Kaplan-Meier method revealed both the overall and disease-free survival models between the subgroups, as shown in Table 5, overall 1-, 3-, and 5-year actual survival rates were comparable between the solitary RFA group and the TACE combined with RFA group (shown in Fig. 2A, P = 0.958); also between the solitary resection group and the TACE combined with resection group (shown in Fig. 2B, P = 0.861); and between the solitary LT group and the TACE combined with LT group (shown in Fig. 2C, P = 0.939). Meanwhile, the overall 1-, 3-, and 5-year tumor-free survival rates also showed no significantly difference between solitary radical therapy group and TACE combined radical therapy group in the three subgroups analysis (Shown in Fig. 2D–F, P > 0.05).

**Figure 3:**
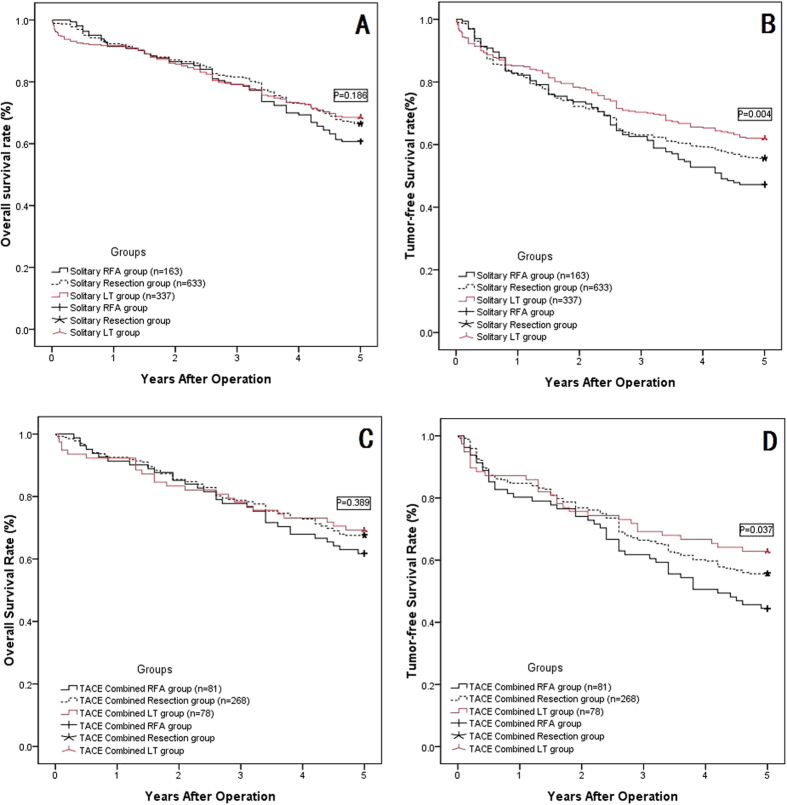
The OSR and TFSR comparison among the RFA, Resection and LT subgroups. (**A**) The OSR comparison among three solitary radical therapy groups: solitarty RFA, resection and LT showed comparable long term OSR (P = 0.186); (**B**) The TFSR comparison among three solitary radical therapy groups: the LT group showed the highest TFSR, followed by Resection group, and RFA is the lowest (p = 0.004); (**C**) The TFSR comparison among three TACE combined radical therapy groups: TACE combined RFA, TACE combined resection and TACE combined LT showed comparable long term TFSR (P = 0.389); (**D**) The TFSR comparison among three TACE combined radical therapy groups: the TACE combined LT group showed the highest TFSR, followed by TACE combined Resection group, and TACE combined RFA is the lowest (p = 0.004).

When we compared the overall survival rate and tumor-free survival rate for the solitary radical groups, i.e., the RFA, resection and LT groups, as shown in Fig. 3A, these three groups showed similar overall survival rates at 1, 3, and 5 years after surgery, and even the solitary LT group showed better survival than the other two groups at 5 years, although this difference did not reach statistical significance (P = 0.186). However, for the tumor-free survival rates at 1, 3, and 5 years after surgery, the solitary LT group showed much better outcomes than did the solitary resection group and the RFA group; at the same time, the solitary resection group also showed better tumor-free survival than the solitary RFA group (shown in Fig. 3B, P = 0.004). Then, we compared the overall survival rate and tumor-free survival rate in the TACE combined subgroups, i.e., the TACE combined with RFA group, TACE combined with resection group, and TACE combined with LT group; these three groups did not show any significantly significant differences in the 1-, 3-, and 5-year overall survival rates (shown in Fig. 3C, P = 0.389). However, for the 1-, 3-, and 5-year tumor-free survival rates, the TACE combined with LT group showed much better outcomes than the other two groups, and the TACE combined with resection group showed much better outcomes than the TACE combined with RFA group (shown in Fig. 3D, P = 0.037).

### Factors contributing to overall survival and tumor-free survival rates after radical therapy

Additional survival analysis was performed and is shown in Tables 6 and 7, including the factors linked to survival, including age, sex, race, BMI, cause of liver disease, preoperative anti-viral therapy, Child class, hemoglobin, platelet, NLR, and AFP levels, tumor number, tumor size, BCLC stage, preoperative TACE, radical therapy, intra-operative blood loss, histological grading, and microvascular invasion. Univariate analysis identified the following prognostic factors predicting poor overall survival: NLR more than 4, AFP more than 400 ng/ml, multiple tumor targets, tumor diameter more than 5 cm, BCLC stage B, poor histological grading, and presence of microvascular invasion. Multivariate Cox regression analysis was performed for these significant factors found on univariate analysis. Multivariate Cox regression analysis showed that NLR greater than 4, multiple tumor targets, BCLC stage B, and poor histological grading represented significant risk factors for the HCC patients’ overall survival after radical therapy.

As shown in Tables 6 and 7, univariate analysis was also performed for the risk or predictive factors for tumor-free survival rate, including an age of 60 years or older, Child class B-C, NLR greater than 4, AFP greater than 400 ng/ml, multiple tumor targets, tumor diameter greater than 5 cm, BCLC stage B, accepting no LT, poor histological grading, and microvascular invasion, which were the 10 significant risk factors contributing to the tumor-free survival rate. The multivariate analysis of these ten factors found to be significant on univariate analysis confirmed that NLR greater than 4, multiple tumor targets, BCLC stage B, accepting no LT, poor histological grading, and microvascular invasion were significant contributors to the tumor-free survival rate.

### Subgroup analysis based on the BCLC stage

**Figure 4:**
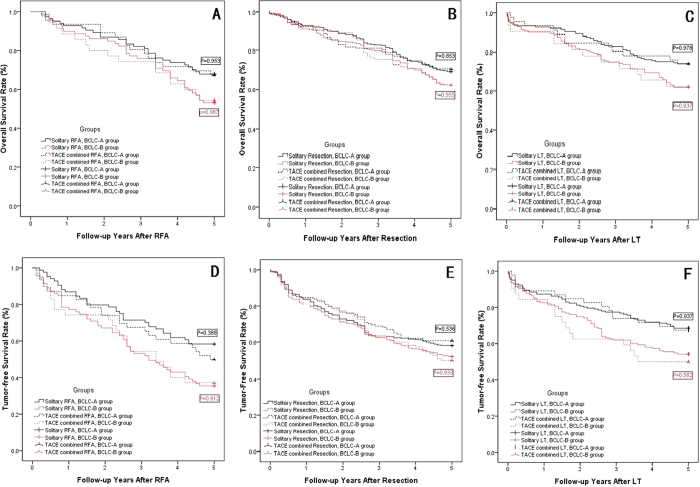
The OSR and TFSR comparison between BCLC-A group and BCLC-B group. (**A**) The OSR comparison between BCLC-A group and BCLC-B group in RFA group: The BCLC-A group and BCLC-B group showed comparable OSR in the solitary RFA subgroup and TACE combined RFA subgroup (P = 0.953 for BCLC-A group, P = 0.987 for BCLC-B group); (**B**) The OSR comparison between BCLC-A group and BCLC-B group in Resection group: The BCLC-A group and BCLC-B group showed comparable OSR in the solitary resection subgroup and TACE combined resection subgroup (P = 0.853 for BCLC-A group, P = 0.955 for BCLC-B group); (**C**) The OSR comparison between BCLC-A group and BCLC-B group in LT group: The BCLC-A group and BCLC-B group showed comparable OSR in the solitary LT subgroup and TACE combined LT subgroup (P = 0.978 for BCLC-A group, P = 0.937 for BCLC-B group); (**D**) The TFSR comparison between BCLC-A group and BCLC-B group in RFA group: The BCLC-A group and BCLC-B group showed comparable TFSR in the solitary RFA subgroup and TACE combined RFA subgroup (P = 0.388 for BCLC-A group, P = 0.912 for BCLC-B group); (**E**) The TFSR comparison between BCLC-A group and BCLC-B group in Resection group: The BCLC-A group and BCLC-B group showed comparable TFSR in the solitary resection subgroup and TACE combined resection subgroup (P = 0.536 for BCLC-A group, P = 0.933 for BCLC-B group); (**F**) The TFSR comparison between BCLC-A group and BCLC-B group in LT group: The BCLC-A group and BCLC-B group showed comparable TFSR in the solitary LT subgroup and TACE combined LT subgroup (P = 0.937 for BCLC-A group, P = 0.582 for BCLC-B group).

**Figure 5:**
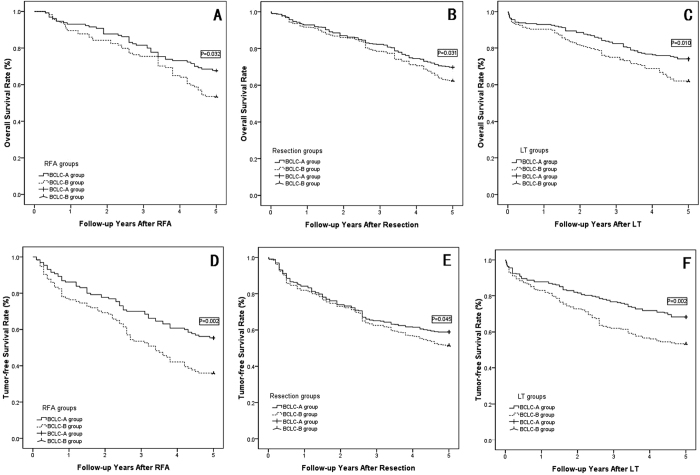
The OSR and TFSR comparison between BCLC-A group and BCLC-B group in the RFA, Resection and LT groups. (**A**) The OSR comparison between BCLC-A and BCLC-B group in RFA group: BCLC-A HCC patients showed higher OSR than BCLC-B patients (p = 0.032); (**B**) The OSR comparison between BCLC-A and BCLC-B group in Resection group: BCLC-A HCC patients showed higher OSR than BCLC-B patients (p = 0.031); (**C**) The OSR comparison between BCLC-A and BCLC-B group in LT group: BCLC-A HCC patients showed higher OSR than BCLC-B patients (p = 0.010); (**D**) The TFSR comparison between BCLC-A and BCLC-B group in RFA group: BCLC-A HCC patients showed higher TFSR than BCLC-B patients (p = 0.002); (**E**) The TFSR comparison between BCLC-A and BCLC-B group in Resection group: BCLC-A HCC patients showed higher TFSR than BCLC-B patients (p = 0.045); (**F**) The TFSR comparison between BCLC-A and BCLC-B group in LT group: BCLC-A HCC patients showed higher TFSR than BCLC-B patients (p = 0.002).

**Figure 6:**
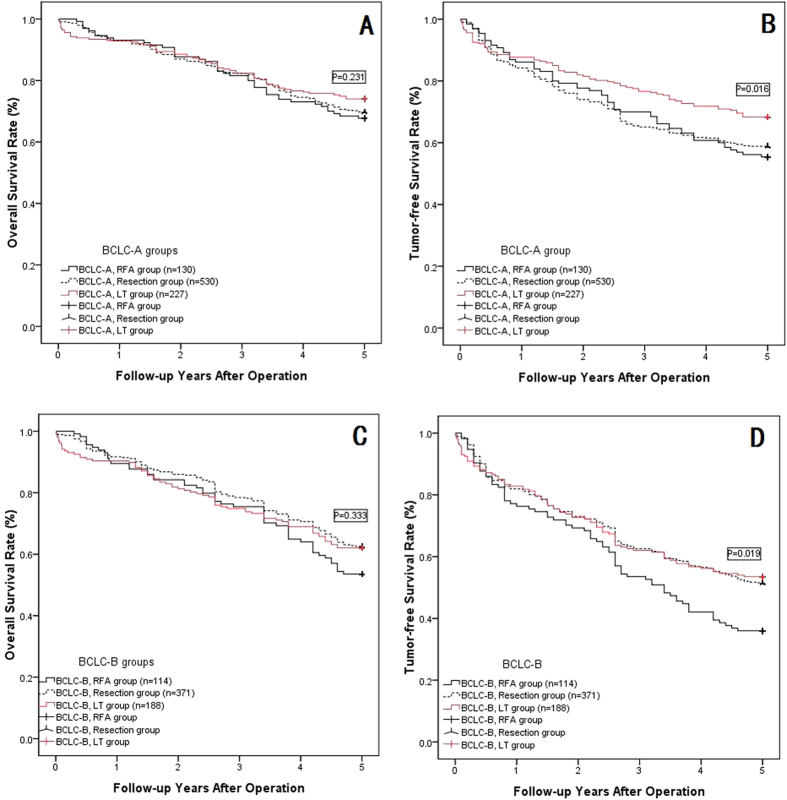
The OSR and TFSR comparison among RFA group, Resection group and LT group for BCLC-A or B HCC patients. (**A**) The OSR comparison among three groups: BCLC-A HCC patients accepted RFA group, Resection group or LT group showed no significantly OSR (P = 0.231); (**B**) The TFSR comparison among three groups: BCLC-A HCC patients accepted LT showed significantly higher TFSR than RFA group and Resection group (P = 0.016); (**C**) The OSR comparison among three groups: BCLC-B HCC patients accepted RFA group, Resection group or LT group showed no significantly OSR (P = 0.333); (**D**) The TFSR comparison among three groups: BCLC-B HCC patients accepted LT or Resection showed significantly higher TFSR than RFA group (P = 0.019).

According to the multivariate analysis, BCLC stage B was a risk factor to overall and tumor-free survival, as shown in Table 8, and the patients in each group were divided into subgroups with BCLC-A or B. The overall survival and tumor-free survival rates were compared in the subgroups: TACE combined with radical therapy (RFA, resection or LT) did not show a significantly better outcome compared with solitary radical therapy in any subgroup analyses (shown in Fig. 4A–E, P > 0.05). However, the BCLC-A HCC patients showed much longer overall and tumor-free survival than did BCLC-B HCC patients in the RFA, resection and LT subgroup analysis (shown in Fig. 5A–E, P < 0.05). The LT group did not show significantly better overall survival rates in the BCLC-A or B groups compared with the resection or RFA groups (shown in Fig. 6A and C, P > 0.05); however, the tumor-free survival rate in the LT group with BCLC-A or B HCCs showed a much better overall survival rate than that of the other two groups (shown in Fig. 6B and D, P < 0.05).

### Causes of death and HCC recurrence

The causes of the death after discharging were comparable between the solitary radical group and TACE combined group. In the sub-group analysis, because of the comparable outcomes for each subgroup in the solitary and TACE combined groups, we analyzed the three subgroups by combining them: the RFA group (244 cases), the resection group (901 cases), and the LT group (415 cases). Tumor recurrence was the leading cause of post-operative death after discharge in all three group, with 86 cases in the RFA group (90.5%), 259 cases in the resection group (90.2%), and 73 cases in the LT group (70.9%). Liver failure was the second most common reason in the RFA group (7 cases, 7.4%) and the resection group (21 cases, 7.3%), however, the second common reason in the LT group was rejection after transplantation and other long-term complications (26 cases, 25.2%). Other reasons included car accident, suicide, and new tumor.

### Literature Review

A total of 43 eligible studies were found to satisfy the inclusion criteria, and the key demographic and clinicopathological data were extracted (Tables 9, 10 and 11). The studies were organized into three subgroups depending on the radical therapy: RFA, resection, and LT. Most of the patients in our included studies were from Asia, the region with the highest prevalence of HCC, including Mainland China, Taiwan, Korea, and Japan.

The majority of the studies reported in Table 9 showed major complication rates ranging from 0.4% to 27.8%. The TACE combined with RFA group showed a better overall survival rate in half of the reports, and only three reports indicated better overall survival rates and tumor-free survival rates in the TACE combined with resection group (shown in Table 10). Pre-LT TACE showed no improvement on overall or tumor-free survival rates in any of the reported studies (shown in Table 11).

## Discussion

The main cause of treatment failure after radical therapies (RFA, resection or LT) for HCC is the high incidence of HCC recurrence. In reports from centers around the world, the 5-year recurrence rates after radical therapies for HCC have ranged from 57% to 100% (shown in Tables 9, 10 and 11), and efforts to prevent and effectively manage the recurrence of HCC are undoubtedly the most important strategies for improving the overall survival with radical treatment for HCC. Many pre-operative strategies have been devised to improve the post-operative overall survival or tumor-free survival, such as pre-operative TACE. The results of the present study showed that performing TACE before radical therapies (RFA, resection or LT) was not beneficial, as radical therapies alone were better for patients with BCLC A-B HCCs, and our study indicated the overall survival rates for TACE combined with radical therapies were comparable with radical therapies alone. The outcomes of tumor recurrence-free survival were also comparable between the groups. In the subgroup analysis, for TACE combined with RFA vs. solitary RFA, TACE combined with resection vs. solitary resection, and TACE combined with LT vs. solitary LT, the overall and tumor-free survival rates were also comparable. Multivariate analyses did not show that pre-operative TACE was a significant prognostic factor for overall or tumor-free survival for HCC patients treated with TACE with radical therapies or radical therapies alone. In our study, although both treatment groups had low and comparable overall or major (grade ≥ III) complication rates, indicating that the rate of complications or major complications after sequential TACE-radical therapy has not been evaluated, when we considered TACE toxicity, 21 patients (4.9%) developed a grade 3 adverse reaction, and 3 patients (0.7%) suffered grade 4 adverse reactions. Our results indicated that additional adverse reactions or even mortality might occur during the pre-operative procedure. Our results were similar to those of the previous studies considered in our literature review.

RFA can result in well-controlled focal thermal injury to a tumor, with minimal morbidity and mortality. Tumor size is considered one of most significant factors for local treatment efficacy^21^. Although RFA has been successfully used to treat small (≤3 cm) HCCs, the local tumor progression rate was higher for tumors that exceeded 3 cm in diameter^7^. Thus, in the present study, we divided our patients into two sub-groups according to the BCLC staging system. Even in the subgroup analysis comparing the BCLC-A and BCLC-B stages, both overall and tumor-free survival were comparable between the solitary RFA group and the TACE combined with RFA group. Some research has indicated that pre-operative TACE might improve the outcomes of RFA, based on the following theory: occlusion of hepatic arterial flow by means of TACE before RFA destroys malignant cells not only in the main tumor but also in daughter tumors, and it reduces the cooling effects of hepatic blood flow on thermal coagulation; and the lipiodol and gelatin sponge particles used in TACE reduce the portal flow around the tumor by filling the peripheral portal vein around the tumor with lipiodol via multiple arterioportal communications^10^. However, as shown in our literature review, many studies have failed to prove the advantages of adjuvant TACE before RFA^14^,^22^,^23^,^24^. There are some potential theories for these negative results: first, it is well known that RFA can be used to treat HCC very successfully compared with liver resection when the diameter is less than 3 cm, as shown in Fig. 3 and in our previous study^25^; however, when the tumor diameter was larger than 3 cm, RFA was not recommended due to the inadequacy of the radiation^26^. Thus, when the diameter was less than 3 cm, the RFA can absolutely irradiate the tumor, so pre-operative strategies such as adjuvant TACE are unnecessary; when the tumor’s diameter was larger than 5 cm, TACE could not change the characteristics of the diameter for radiation. Second, although TACE before RFA could detect and control a daughter or satellite tumor, TACE could not destroy these targets; rarely, a study reported a cure of HCC with TACE^27^, but our previous research proved that TACE could not cure HCC^28^. Finally, pre-operative TACE can lead to perihepatic adhesions, which can result in more difficult surgical procedures and greater intra-operative blood loss, particularly with open access, and these complications could reduce the effectiveness of RFA for HCC^29^. Combined TACE and radical therapy can lead to increased patient discomfort, prolonged hospital stays, and, although we did not formally assess it, an obvious increase in costs. Therefore, adjuvant pre-RFA TACE should not be recommended for HCC patients.

Although some case-control studies have shown that hepatectomy with adjuvant TACE efficaciously and safely improved survival outcomes compared with hepatectomy alone^9^,^30^,^31^, many case-control studies did not show that adjuvant TACE could reduce the incidence of recurrence or prolonging survival in HCC^11^,^13^,^32^,^33^,^34^. Two randomized, controlled trails on neoadjuvant TACE used before partial hepatectomy for HCC showed that neoadjuvant therapy had no impact on tumor-free or overall survival, compared with the control group^11^,^35^. Further, in Wu *et al*.’s study^36^, the 52 randomized patients with resectable HCC showed decreased overall survival and a higher extrahepatic recurrence rate compared with the control group. In agreement with these previous studies, we found that preoperative TACE did not improve overall or tumor-free survival after resection of HCC.

The arguments against the use of neoadjuvant TACE include^11^: the associated complications with preoperative TACE, such as perihepatic adhesions, rendering liver resection more difficult; liver function impairment and the increased risk of liver failure; a delay in definitive surgery, causing some resectable tumors to become unresectable; increased difficulty in future transarterial treatment for recurrent HCC as a result of the development of collateral neoplastic feeding vessels after embolization of hepatic arteries; and partial tumor necrosis induced by adjuvant TACE causing the remaining tumor cells to become less firmly attached and more likely to be dislodged into the bloodstream during hepatic resection. Further, in the present study, the TACE combined with LT group showed worse liver function when they accepted LT, so we should pay attention to this liver function impairment, particularly in RFA and resection patients. At the same time, perihepatic adhesions were much more common in the TACE combined groups: 81.5% in the TACE combined with RFA group; 76.5% in the TACE combined with resection group; and 70.5% in the TACE combined with LT group. Even with more perihepatic adhesions in the TACE combined with resection group than in the solitary resection group, the TACE combined with resection group required a shorter operative time but suffered more post-operative complications. Further, the main reason for the shorter operative time might have been the occlusion of hepatic arterial flow in both the tumor and the peritumor tissue; at the same time, the lipiodol and gelatin sponge particles used in TACE reduce the portal flow around the tumor by filling the peripheral portal vein around the tumor with lipiodol via multiple arterioportal communications^10^. Pre-resection TACE reduced the tumor mass, thus making resection easier and rendering the tumor less vascular, causing the uninvolved liver to hypertrophy to allow for safer resection; these factors might have reduced the intra-operative blood loss and made parenchymal dissectioneasier with a shorter operative time. Although the difference inblood loss between the two groups did not reach a statistically significantly difference, there was a trend toward less blood loss in the TACE combined with resection group (P = 0.084), and a larger cohort might prove this. The vessels and bile duct are the two key points in the parenchymal dissection procedure; TACE could reduce intra-operative blood loss, leading to a more rapid dissection procedure that ignores the bile duct. Thus, in the present study, there were more complications in the TACE combined with resection group (36.9% vs. 29.4%, P = 0.014), primarily a higher rate of bile leakage (15.3% vs. 8.8%, P = 0.004), which were generally classified as grade I or II; however, the adverse complications did not show a significant difference.

Despite the high risk to living donors and the shortage of liver grafts, LT should be considered the first choice for the small HCCs (within the Milan or UCSF criteria), particularly for patients with liver cirrhosis. LT offers the theoretical advantages of removing the tumor and the risk of the organ developing future malignancy; most importantly, some satellites or cells in the liver can also be removed^37^. However, one of the most common characteristics of LT, compared with RFA or resection, is the waiting time required for the deceased or living donor liver graft. Llovet et al reported that there can be increased tumor development during the waiting time for LT, and up to 23% patients are missed because of tumor progression or death from liver disease, which decreases the potential benefit of LT for HCC^38^. TACE was introduced for LT candidates waiting for liver grafts. However, when LT can be performed, is TACE still necessary? In the present study, all of the TACE procedures were performed 2 weeks before LT, so TACE did not extend the waiting period and was only attempted to improve the outcomes of LT. Although we did not observe an increase in postoperative mortality or morbidity from TACE and LT, we were not able to demonstrated a significant benefit for the overall survival or tumor-free survival rate after LT in patients who previously underwent TACE, and this finding coincided with larger series in our literature review^39^,^40^,^41^,^42^,^43^,^44^. The main reason for this negative result might have been the characteristics of LT, including removal of the whole liver. Therefore, pre-LT TACE for the theoretical advantages of TACE before RFA or resection, such as satellite target control or reducing the portal or artery flow, was not found. However, pre-LT TACE could serve as a selective method for HCC patients^27^,^45^: characteristics of tumor response to TACE are reliably recognized and allow for the identification of suitable patients for transplantation. Selective TACE requires a long time to observe the response to TACE, not the two weeks in our present study. In our future work, we will examine this aspect.

When we compared the effectiveness of these three radical groups, our results showed comparable 1-, 3- and 5-year overall survival rates in the three groups but better long-term tumor-free survival in the LT group than in the resection group and RFA group, as shown in Fig. 3. In the BCLC subgroup analysis, similar outcomes were observed, as shown in Fig. 6. In BCLC stage A HCC patients, LT can provide better local tumor control than either RFA or resection; however, in BCLC stage B HCC patients, LT provided similar local tumor control to resection but was better than RFA, primarily because the BCLC stage B HCCs did not meet the Milan criteria or UCSF criteria, which were considered the inclusion criteria for LT^15^,^16^. The treatment outcomes for HCC patients were affected by multiple variables, including tumor burden, the Child-Pugh score of liver function reserve, and the performance status of the patients^5^. The Barcelona Clinic Liver Cancer (BCLC) classification staging system considered these 3 variablesin 1999^46^,^47^. The BCLC staging system has been validated by several groups in Europe and the United States^48^,^49^. In addition to estimating prognosis, the main advantage of the BCLC staging system is the establishment of links between staging and treatment indications^50^. The BCLC staging system recommends different treatment options for each stage of the disease. According to this staging system, radical therapy is indicated for the early stages (LT, resection, RFA), loco-regional therapy (TACE) is recommended for the intermediate stage, and oncologic treatment with a multikinase inhibitor (sorafenib) is recommended for the advanced stage (with portal invasion or metastasis); in the end stage, only supportive care is recommended^46^. The present study showed a difference in long-term outcomes between patients with BCLC-A and BCLC-B HCCs when they accepted radical therapies. Due to the much lower overall survival or tumor-free survival rates in the BCLC-B HCC patients compared with the BCLC-A HCC patients, these three radical therapies can be recommended to patients with BCLC-A HCCs, but with extreme caution for BCLC-B HCCs; our results were consistent with those from the literature^51^,^52^.

This study was limited by a possible selection bias resulting from the comparison of these non-randomized groups and retrospective profiles; however, this was a single-center experience, and the results might not be generalizable; However, our large group analysis and subgroup analysis should serve to strengthen the conclusion that the combination of TACE does not offer an improvement over solitary radical therapy alone.

In conclusion, preoperative adjuvant TACE prolonged neither long-term overall survival nor tumor-free survival in patients who accepted RFA, resection or LT. Thus, despite its relatively safety and feasibility, we cannot recommend preoperative adjuvant TACE as a routine procedure before radical therapy in HCC patients. LT should remain the first choice for BCLC-A HCC patients.

## Additional Information

**How to cite this article:** Jianyong, L. *et al*. Preoperative adjuvant transarterial chemoembolization cannot improve the long term outcome of radical therapies for hepatocellular carcinoma. *Sci. Rep.* **7**, 41624; doi: 10.1038/srep41624 (2017).

**Publisher's note:** Springer Nature remains neutral with regard to jurisdictional claims in published maps and institutional affiliations.

## Figures and Tables

**Table 1:** Main inclusion/exclusion criteria of the study.

**Inclusion criteria**
Primary hepatocellular carcinoma
Targets with no previous treatment
Liver cirrhosis classified as Child class A or B
BCLC-HCC stage 0 or A
Accepting RFA, resection or LT
**Exclusion criteria**
Presence of macro-vascular invasion
Present of extrahepatic target
Severe impairment of another organ
Metastatic hepatic malignancies
Child class C
**Gastrointestinal hemorrhage in the past month**
Gallbladder carcinoma or extrahepatic primary biliary carcinoma
Intrahepatic cholangiocarcinoma
Metastatic liver disease
Rupture of HCC
Loss to follow-up

HCC: hepatocellular carcinoma; RFA: Radiofrequency ablation; LT: liver transplantation.

**Table 2:** Baseline demographic and tumor characteristics compared between the solitary radical groups and the combined TACE group.

	RFA	TACE + RFA	P value	Resection	TACE + resection	P value	LT	TACE + LT	P value
Patient number	163	81		633	268		337	78	
Age (year)	51.1 ± 11.8	56.2 ± 12.1	0.002	52.0 ± 12.8	51.4 ± 13.3	0.647	52.2 ± 12.6	53.8 ± 12.1	0.325
Sex (M/F)	112/51	53/28	0.607	466/167	186/82	0.196	230/107	53/25	0.959
Race (Han/Tibetan/other)	152/8/3	75/5/1	0.861	595/31/7	253/11/4	0.824	315/34/5	73/4/0	0.970
BMI (kg/m^2^)	23.4 ± 2.4	23.3 ± 2.5	0.838	23.6 ± 2.3	23.6 ± 2.2	0.854	23.6 ± 2.3	23.5 ± 2.2	0.914
Underlying liver disease (HBV/HCV/negative)	135/10/18	70/3/8	0.831	535/32/66	225/19/24	0.226	282/19/36	65/6/7	0.461
Pre-operative anti-viral therapy (yes/no)	82/79	51/30	0.091	391/241	167/101	0.913	195/142	46/32	0.858
Child class (A/B/C)	94/69	46/35	0.928	356/277	159/109	0.392	186/107/44	31/25/22	0.002
Hemoglobin (g/l)	125.8 ± 26.9	124.9 ± 24.1	0.798	128.2 ± 24.6	131.3 ± 22.1	0.086	131.5 ± 25.7	127.6 ± 22.2	0.230
Platelets (x10^9^/l)	136.5 ± 92.3	163.9 ± 144.7	0.096	146.9 ± 85.2	140.7 ± 81.4	0.355	135.6 ± 82.7	123.9 ± 87.0	0.306
NLR (<4/≥4)	90/73	46/35	0.816	334/299	139/129	0.805	174/163	38/40	0.643
Tumor size(cm)	5.6 ± 1.9	5.3 ± 2.5	0.304	5.3 ± 2.3	5.4 ± 2.1	0.278	5.6 ± 2.2	5.9 ± 2.0	0.362
Tumor number (1/2/3/multiple)	20/58/54/31	26/24/25/6	0.101	163/305/129/36	54/138/71/5	0.230	68/150/100/19	14/42/22/0	0.390
AFP level (ng/ml)	907.8 ± 3251.6	1216.2 ± 4570.2	0.557	1089.4 ± 7450.7	1552.2 ± 10956.0	0.475	1532.5 ± 9982.0	804.1 ± 3780.7	0.535
BCLC stage (0-A/B)	84/79	46/35	0.439	372/261	158/110	0.958	181/156	46/32	0.400

BCLC: Barcelona Clinic Liver Cancer; M: male; F: female; BMI: body mass index; HBV: hepatitis B virus; HCV: hepatitis C virus; AFP: alpha fetoprotein; NLR: neutrophil-lymphocyte ratio.

**Table 3:** Adverse events following TACE.

Adverse reactions (%)	Grade 1	Grade 2	Grade 3	Grade 4
Nausea/emesis	212 (49.6%)	18 (4.2%)	2 (0.5%)	0
Fever	165 (38.6%)	35 (8.2%)	3 (0.7%)	0
Pain in upper quadrant	179 (41.9%)	46 (10.8%)	0	0
Ischemic liver function destroyed	337 (78.9%)	24 (5.6%)	4 (0.9%)	1 (0.2%)
Femoral artery pseudoaneurysm	0	0	2 (0.5%)	0
Thrombosis of superficial femoral artery	0	0	1 (0.2%)	0
Spontaneous bacterial peritonitis	0	0	2 (0.5%)	1 (0.2%)
Allergy	0	5 (1.2%)	2 (0.5%)	0
Sepsis	0	0	2 (0.5%)	1 (0.5%)
Acute renal failure	0	0	3 (0.7%)	0

**Table 4:** Operative variables and perioperative outcomes comparison.

	RFA	TACE + RFA	P value	Resection	TACE + resection	P value	LT	TACE + LT	P value
Patient number	163	81		633	268		337	78	
Blood loss (ml)	101.8 ± 111.4	103.1 ± 105.9	0.933	384.0 ± 214.1	357.8 ± 191.4	0.084	1973.6 ± 803.0	1990.4 ± 809.8	0.868
Perioperative blood transfusion (%)	3(1.8%)	0(0%)	0.220	82(13.0%)	32(11.9%)	0.676	303(90.0%)	69(88.5%)	0.705
Operative time (h)	2.7 ± 0.9	2.6 ± 0.8	0.328	4.3 ± 1.7	3.9 ± 1.3	<0.001*	9.6 ± 12.2	10.1 ± 13.3	0.751
Perihepatic adhesions (%)	11(6.7%)	66(81.5%)	<0.001*	31(4.9%)	205(76.5%)	<0.001*	20(5.9%)	55(70.5%)	<0.001*
Hospital stay (days)	5.0 ± 1.3	4.8 ± 1.3	0.320	5.8 ± 2.0	6.0 ± 2.9	0.127	33.0 ± 11.2	32.5 ± 10.7	0.701
ICU stay (%)	1(0.6%)	0(0%)	0.613	73(11.5%)	29(10.8%)	0.758	337(100%)	78(100%)	>0.05
In-hospital morbidity (%)	1(0.6%)	0(0%)	0.481	8(1.3%)	3(1.1%)	0.857	28(8.3%)	7(8.9%)	0.849
Post-operative complications (Clavien system)	37(22.7%)	19(23.5%)	0.816	186(29.4%)	99(36.9%)	0.014*	163(48.4%)	40(51.2%)	0.465
Grade I	19	8		110	48		42	10	
Grade II	13	7		41	28		55	18	
Grade IIIa	2	2		9	12		20	2	
Grade IIIb	2	2		8	5		9	1	
Grade Iva	0	0		7	3		6	1	
Grade IVb	0	0		3	0		3	1	
Grade V	1	0		8	3		28	7	
Histological grading									
Good	59	25	0.737	187	71	0.61	105	28	0.351
Moderate	45	25		219	91		112	26	
Poor	46	21		227	95		120	21	
Unknown	13	10		0	11		0	3	

ICU: intensive care unit; Postoperative complications were graded using the Clavien-Dindo classification.

**Table 5:** 1-, 3-, and 5-year overall and tumor-free survival rate comparison.

	1-, 3-, and 5-year overall survival rate (%)			P value	1-, 3-, and 5-year tumor-free survival rate (%)			P value
	1-year	3-year	5-year		1-year	3-year	5-year	
Solitary radical therapy group (n = 1133)	92.0%	80.5%	66.2%	0.955	88.3%	65.1%	56.3%	0.746
TACE combined group (n = 427)	92.3%	78.5%	66.7%		84.3%	66.3%	54.8%	
Solitary RFA group (163)	91.4%	79.1%	60.7%	0.958	82.8%	62.6%	47.2%	0.696
TACE combined with RFA group (81)	91.4%	77.8%	61.7%		80.2%	61.7%	44.4%	
Solitary resection group (633)	92.3%	81.5%	66.5%	0.861	82.6%	63.0%	55.6%	0.678
TACE combined with resection group (268)	92.5%	78.7%	67.5%		84.7%	66.4%	56.3%	
Solitary LT group (337)	91.7%	79.2%	68.5%	0.939	85.2%	70.3%	62.0%	0.782
TACE combined with LT group (78)	92.3%	78.2%	69.2%		87.2%	70.5%	60.3%	

TACE: transarterial chemoembolization; RFA: Radiofrequency ablation; LT: liver transplantation.

**Table 6:** Univariate analyses contributing to overall survival and tumor-free survival rates after radical therapy.

Variables	N	Overall survival rate	Tumor-free survival rate
		P value	P value
Age ≥ 60 (yes/no)	451/1109	0.279	0.011*
Sex (M/F)	1100/460	0.861	0.922
Race (Han/other)	1463/97	0.966	0.920
BMI ≥ 28 (yes/no)	81/1479	0.848	0.532
Causes of liver diseases			
HBV	1312		
HCV	89	0.569	0.322
No	159	0.190	0.122
Pre-operative anti-viral therapy (yes/no)	932/628	0.329	0.316
Child class (A/B-C)	872/688	0.134	0.044*
Hemoglobin ≥ 120 g/l (yes/no)	1028/532	0.795	0.354
Platelet ≥ 100*10^9/l (yes/no)	862/698	0.416	0.597
NLR ≥ 4 (yes/no)	739/821	0.047*	0.008*
AFP ≥ 400ng/ml (yes/no)	587/973	0.001	0.029*
Tumor number			
1	345		
2–3	1118	0.156	0.098
Multiple	97	<0.001*	<0.001*
Tumor diameter ≥5 cm (yes/no)	1016/544	<0.001*	0.001*
BCLC stage (0-A/B)	887/673	<0.001*	<0.001*
Preoperative TACE (yes/no)	427/1133	0.864	0.593
Radical therapy			
RFA	244		
Resection	901	0.202	0.005*
LT	415	0.166	0.001*
Intra-operative blood loss ≥400 ml (yes/no)	873/687	0.809	0.150
Histological grading			
Good	475		
Moderate	518	0.155	0.211
Poor	530	0.028*	0.010*
Unknown	37	0.013*	0.021*
Microvascular invasion (yes/no)	692/868	0.021*	0.001*

BCLC: Barcelona Clinic Liver Cancer; M: male; F: female; BMI: body mass index; HBV: hepatitis B virus; HCV: hepatitis C virus; AFP: alpha fetoprotein; NLR: neutrophil-lymphocyte ratio; TACE: transarterial chemoembolization; RFA: Radiofrequency ablation; LT: liver transplantation.

**Table 7:** Multivariate analyses contributing to overall survival and tumor-free survival rates after radical therapy.

Variables	Hazard ratio	95% CI	value
Prognostic factors for overall survival			
NLR ≥ 4	1.262	1.121–1.579	0.026*
AFP ≥ 400 ng/ml	1.382	0.945–2.453	0.124
Tumor number			
1			
2–3	1.462	0.823–1.982	0.213
Multiple	1.232	1.087–1.562	0.002*
Tumor diameter ≥ 5 cm	1.081	0.909–1.172	0.542
BCLC stage B	1.452	1.272–1.679	<0.001*
Histological grading			
Good			
Moderate	1.321	1.192–1.729	0.439
Poor	1.782	1.682–1.913	0.031*
Unknown	0.152	0.022–0.242	<0.001
Microvascular invasion	1.254	1.121–1.359	0.352
Prognostic factors for tumor-free survival			
Age ≥ 60	0.928	0.782–1.112	0.254
Child class (A/B-C)	1.212	1.021–1.438	0.452
NLR ≥ 4	1.453	1.212–1.552	0.002*
AFP ≥ 400 ng/ml	1.211	0.972–1.432	0.110
Tumor number			
1			
2–3	1.132	0.893–1.287	0.102
Multiple	1.328	1.011–1.542	0.002*
Tumor diameter ≥ 5 cm	1.243	0.996–1.326	0.137
BCLC stage B	1.603	1.226–2.902	<0.001*
Radical therapy (RFA/resection/LT)			
RFA			
Resection	0.611	0.404–0.832	<0.001*
LT	0.521	0.336–0.753	<0.001*
Histological grading (good/moderate/poor)			
Good			
Moderate	1.321	1.107–1.543	0.087
Poor	1.452	1.190–1.574	<0.001*
Unknown	0.212	0.101–0.437	<0.001*
Microvascular invasion	1.328	1.212–1.453	0.003*

BCLC: Barcelona Clinic Liver Cancer; BMI: body mass index; AFP: alpha fetoprotein; NLR: neutrophil-lymphocyte ratio; TACE: transarterial chemoembolization; RFA: Radiofrequency ablation; LT: liver transplantation.

**Table 8:** 1-, 3-, and 5-year overall and tumor-free survival rate comparison according to the BCLC staging system.

	1-, 3-, and 5-year overall survival rate (%)			P value	1-, 3-, and 5-year tumor-free survival rate (%)			P value
	1-year	3-year	5-year		1-year	3-year	5-year	
BCLC-A, solitary RFA (84)	92.9%	82.1%	67.9%	0.953	86.9%	71.4%	58.3%	0.388
BCLC-A, TACE combined with RFA (46)	93.5%	80.4%	67.4%		84.8%	67.4%	50.0%	
BCLC-B, solitary RFA (79)	89.9%	75.9%	53.2%	0.987	78.5%	53.2%	35.4%	0.912
BCLC-B, TACE combined with RFA (35)	88.6%	74.3%	54.3%		74.3%	54.3%	37.1%	
RFA, BCLC-A (130)	93.1%	81.5%	67.7%	0.032*	86.2%	70%	55.4%	0.002*
RFA, BCLC-B (114)	89.5%	75.4%	53.5%		77.2%	53.5%	36.0%	
BCLC-A, solitary resection (372)	93.0%	82.8%	69.4%	0.536	83.9%	63.4%	58.1%	0.853
BCLC-A, TACE combined with resection (158)	92.3%	81.0%	70.9%		84.8%	69.0%	60.8%	
BCLC-B, solitary resection (261)	91.2%	79.7%	62.5%	0.933	80.8%	62.5%	52.1%	0.955
BCLC-B, TACE combined Resection (110)	92.7%	75.5%	62.7%		84.5%	62.7%	50.0%	
Resection, BCLC-A (530)	92.8%	82.3%	69.8%	0.031	84.2%	65.1%	58.9%	0.045*
Resection, BCLC-B (371)	91.6%	78.4%	62.5%		81.9%	62.5%	51.5%	
BCLC-A, solitary LT (181)	92.8%	82.9%	74.0%	0.978	87.3%	77.3%	68.6%	0.937
BCLC-A, TACE combined with LT (46)	93.5%	80.4%	73.9%		89.1%	76.1%	67.4%	
BCLC-B, solitary LT (156)	90.4%	75.0%	62.2%	0.937	82.7%	62.2%	54.5%	0.528
BCLC-B, TACE combined with LT (32)	90.6%	75.0%	62.5%		84.4%	62.5%	50.0%	
LT, BCLC-A (227)	93.0%	82.4%	74.0%	0.010*	87.7%	77.1%	68.3%	0.002*
LT, BCLC-B (188)	90.4%	75.0%	62.2%		83.0%	62.2%	53.7%	

BCLC: Barcelona Clinic Liver Cancer; TACE: transarterial chemoembolization; RFA: Radiofrequency ablation; LT: liver transplantation.

**Table 9:** Recent reports concerning the use of preoperative TACE on HCCs patients who accepted RFA.

First author	Country	Published Year	Recruitment year	Patient number	Inclusion criteria	Treatment protocol	Patient number	Major complication rate	Response rate (complete/partial)	1-, 3-, and 5-year overall survival rates (%)			P value
Kim^14^	Korea	2012	2001–2008	314	2–3 cm	RFA	231	0.4%	99%	93%	73%	53%	P = 0.545
						TACE + RFA	83	1.2%	99%	93%	72%	63%	
Kim^7^	Korea	2011	2000–2010	123	3.1–5.0 cm	RFA	66	3%	94%	—	—	23%	P < 0.05
						TACE + RFA	57	0%	98%	—	—	49%	
Morimoto^22^	Japan	2010	2005–2009	42	3.1–5.0 cm	RFA	18	27.8%	100%	89%	80%	—	P = 0.369
						TACE + RFA	19	5.3%	100%	100%	93%	—	
Kim^23^	Korea	2013	2008–2010	84	2–5 cm	RFA	47	14.9%	100%	95.7%	84.3%	—	P = 0.631
						TACE + RFA	37	2.7%	100%	97.3%	78.4%	—	
Cheng^53^	China	2008	2001–2004	196	Larger than 3 cm	RFA	100	5%	37%/32%	67%	32%	8%	P < 0.01
						TACE + RFA	96	10.4%	55%/24%	83%	55%	31%	
Peng^10^	China	2012	2002–2006	139	Less than 5 cm	RFA	70	2.8%	100%	82%	47%	36%	P = 0.037
						TACE + RFA	69	2.9%	100%	94%	69%	46%	
Yang^54^	China	2009	2000–2007	103	Recurrence after resection	RFA	37	2.7%	100%	73.9%	51.1%	28.0%	P < 0.05
						TACE + RFA	31	3.2%	100%	88.5%	64.6%	44.3%	
Shibata^24^	Japan	2009	2003–2007	89	≤3 cm	RFA	43	2%	100%	100%	84.5%	74%	P = 0.515
						TACE + RFA	46	2%	100%	100%	84.8%	72.7%	
Yang^55^	China	2008	2004–2006	78	No limit	RFA	12	—	47.8%	57.6%	52.3%	—	P < 0.01
						TACE + RFA	31	—	88.6%	81.20%	77.1%	—	
Present study	China	present	2005–2009	244	BCLC 0-A/B	RFA	81	3.1%	100%	91.4%	79.10%	60.7%	0.958
						TACE + RFA	163	4.9%	100%	91.4%	77.8%	61.7%	

BCLC: Barcelona Clinic Liver Cancer; TACE: transarterial chemoembolization; RFA: Radiofrequency ablation; LT: liver transplantation.

**Table 10:** Recent reports concerning the use of preoperative TACE in HCC patients who accepted resection.

First author	Country	Published year	Recruitment year	Patient number	Inclusion criteria	Treatment protocol	Patient number	Complication rate	Overall survival rate (%)			P Value	Tumor-free survival rate (%)			P value
									1-year	3-year	5-year		1-year	3-year	5-year	
Zhong^9^	China	209	2001–2004	115	Stage IIIA	Resection	58	34.5%	56.5%	19.4%	17.5%	P = 0.0048	14.0%	3.5%	1.7%	P = 0.004
						Combined	57	31.6%	80.7%	33.3%	22.8%		29.7%	9.3%	9.3%	
Zhou^11^	China	2009	2000–2003	108	≥5 cm	Resection	56	21.4%	69.6%	32.1%	21.1%	P = 0.679	39.2%	21.4%	8.9%	P = 0.372
						Combined	52	31.9%	73.1%	40.4%	30.7%		48.9%	25.5%	12.8%	
Lee^13^	Taiwan	2009	2000–2006	350	TNM I-III	Resection	236	NA	89%	73%	59%	P = 0.025	66%	44%	32%	P = 0.955
						Combined	114	—	81%	57%	47%		60%	49%	40%	
Nishikawa^32^	Japan	2013	2004–2012	235	Resectable	Resection	125	NA	94.9%	79.0%	57.8%	P = 0.674	73.3%	48.9%	33.2%	P = 0.062
						Combined	110	—	87.4%	76.0%	62.5%		73.3%	29.4%	16.3%	
Shi^33^	Taiwan	2014	1996–2009	11079	Resectable	Resection	10431	NA	82.8%	61.0%	48.1%	P = 0.777	52.9%	30.2%	20.7%	P = 0.777
						Combined	648	—	83.7%	63.5%	61%		55.6%	34.2%	20.3%	
Kaibori^34^	Japan	2012	2004–2007	124	Resectable	Resection	43	NA	83%	60%	56%	P = 0.412	53%	32%	—	P = 0.660
						Combined	81	—	88%	75%	47%		65%	27%	—	
Yamashita^30^	Japan	2012	1995–2008	137	≥5 cm	Resection	95	26.2%	—	—	43%	P = 0.02	—	—	37%	P = 0.04
						Combined	42	23.3%	—	—	57%		—	—	43%	
Kang^31^	Korea	2010	1997–2007	96	Resectable	Resection	64	NA	97%	83%	45%	P = 0.11	77%	58%	32%	P = 0.001
						Combined	32	—	78%	60%	26%		58%	36%	7%	
Present study	China	Present	2005–2009	901	BCLC0-A/B	Resection	633	29.4%	92.3%	81.5%	66.5%	P = 0.861	82.6%	63.0%	55.6%	P = 0.678
						Combined	268	36.9%	92.5%	78.7%	67.5%		84.7%	66.4%	56.3%	

BCLC: Barcelona Clinic Liver Cancer; TACE: transarterial chemoembolization; RFA: Radiofrequency ablation; LT: liver transplantation.

**Table 11:** Recent reports concerning the use of preoperative TACE in HCC patients who accepted liver transplantation.

First author	Country	Published year	Recruitment Year	Patient number	Inclusion criteria	Treatment protocol	Patient number	Overall survival rate (%)			P Value	Tumor-free survival rate (%)			P value
								1-year	3-year	5-year		1-year	3-year	5-year	
Eswaran^39^	USA	2012	1999–2008	39	None	LT	7	92%	77%	77%	P = 0.28	—	—	—	—
						TACE + LT	28	100%	100%	82%		—	—	—	—
Seehofer^40^	Germany	2012	1989–2008	177	USCF	LT	106	87%	76%	67%	P = 0.522	—	—	—	—
						TACE + LT	71	92%	80%	73%		—	—	—	—
Schaudt^41^	Germany	2009	1995–2005	27	None	LT	12	92%	92%	61%	P = 0.5	—	—	—	—
						TACE + LT	15	100%	93%	82%		—	—	—	—
Decaens^42^	France	2005	1995–1998	200	None	LT	100	82%	68%	59.4%	P > 0.05	83%	75%	64.1%	P > 0.05
						TACE + LT	100	85%	78%	59.3%		80%	74%	69.3%	
Yao^56^	USA	2005	1999–2002	90	T2–3	LT	41	—	—	—		91.5%	80.6%	80.6%	P = 0.049
						TACE + LT	85	—	—	—		96.4%	93.8%	93.8%	
Perez^43^	Spain	2005	1986–2001	46	Okuda I-III	LT	28	77.2%	58.7%	38.1%	P = 0.56	68.2%	54.2%	39.5%	P = 0.8
						TACE + LT	18	83.3%	60.5%	60.5%		77.8%	54.3%	54.3%	
Oldhafer^44^	Germany	1998	1993–1996	42	UICC I-IV	LT	21	61.5%	53.9%	45.2%	P > 0.05	85.2%	70.3%	62.0%	0.782
						TACE + LT	21	60.8%	48.4%	48.4%		87.2%	70.5%	60.3%	
Present study	China	Present	2005–2009	415	BCLC 0-A /B	LT	337	91.7%	79.2%	68.5%	0.939	85.2%	70.3%	62.0%	0.783
						TACE + LT	78	92.3%	78.2%	69.2%		87.2%	70.5%	60.2%	

BCLC: Barcelona Clinic Liver Cancer; TACE: transarterial chemoembolization; RFA: Radiofrequency ablation; LT: liver transplantation; UCSF: University of California.
